# Exposome, Molecular Pathways and One Health: The Invertebrate *Caenorhabditis elegans*

**DOI:** 10.3390/ijms23169084

**Published:** 2022-08-13

**Authors:** Anna von Mikecz

**Affiliations:** IUF—Leibniz Research Institute for Environmental Medicine GmbH, Auf’m Hennekamp 50, 40225 Duesseldorf, Germany; mikecz@tec-source.de

**Keywords:** big data, *C. elegans*, exposome, mercury, neurotoxins, nanoparticles, nanotoxicology, one health

## Abstract

Due to its preferred habitats in the environment, the free-living nematode *Caenorhabditis elegans* has become a realistic target organism for pollutants, including manufactured nanoparticles. In the laboratory, the invertebrate animal model represents a cost-effective tool to investigate the molecular mechanisms of the biological response to nanomaterials. With an estimated number of 22,000 coding genes and short life span of 2–3 weeks, the small worm is a giant when it comes to characterization of molecular pathways, long-term low dose pollutant effects and vulnerable age-groups. Here, we review (i) flows of manufactured nanomaterials and exposition of *C. elegans* in the environment, (ii) the track record of *C. elegans* in biomedical research, and (iii) its potential to contribute to the investigation of the exposome and bridge nanotoxicology between higher organisms, including humans. The role of *C. elegans* in the one health concept is taken one step further by proposing methods to sample wild nematodes and their molecular characterization by single worm proteomics.

## 1. Introduction

Current estimations predict that approximately 350,000 chemicals are in production and usage globally [1]. Nanomaterials such as nano silica have been industrially produced in considerable varieties for decades and remain widely applied in products ranging from varnishes or car tires to additives in pesticides [2]. Corresponding environmental flows into environmental sinks have been modelled for the EU with median nano silica concentrations of 0.12 μg/L in surface waters and an annual increase of 0.43 mg/kg in sediments. Other manufactured nanoparticles, such as nano silver (Ag), nano titanium dioxide (TiO_2_), and nano plastics, likewise represent contaminants of emerging concern that distribute to air, freshwater habitats, marine habitats, sediments, and soil [3,4]. Nano plastics likely represent secondary nanoparticles arising as fragmentation products from microplastics that meanwhile flow to and are sampled from virtually all areas of our planet, including the arctic and deep sea. Notably, applications of manufactured nanomaterials with a significant potential for environmental distribution include their intentional use in agriculture and water treatments.

In order to keep up with the pace of nanotechnology and the distribution of manufactured nanomaterials into the environment, new and efficient methodologies are needed for both the investigation of biological responses to nanoparticles and the practice of safe nanotechnological applications. This review focuses on the contribution of the invertebrate model organism *Caenorhabditis elegans* to investigations concerning the biological effects of manufactured nanomaterials. *C. elegans* is presented as a relevant target organism for nanomaterials and an animal model for neurotoxicology and life span-resolved studies, as well as the analysis of molecular pathways of neurodegeneration. With its ease of maintenance in the laboratory and options for medium to high throughput *C. elegans* is optimally suited to inform the concepts of the exposome and one health.

## 2. *C. elegans* in the Environment

**Figure 1:**
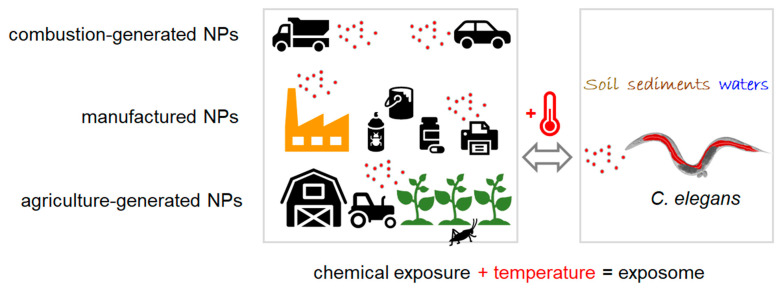
The first encounter of wild *C. elegans* with different nanomaterials (schematic). Combustion-generated, manufactured and agriculture-generated nanoparticles represent chemical stressors. Nanomaterials flow to the environmental compartments soil, sediments and surface waters. Ambient temperature constitutes an additional non-chemical stressor that adds—together with the chemical exposure—to the exposome. *C. elegans*, *Caenorhabditis elegans*; NPs, nanoparticles.

The nematode roundworm *C. elegans* is used in the laboratory as a major model organism for biomedical research and occurs in the wild as a bacterivore that lives on rotting plant material. Probabilistic and dynamic modelling of pollutant flows in technical and environmental compartments together with the habitats of wild *C. elegans* predicts where the nematode is exposed to xenobiotics, such as manufactured nanomaterials (Figure 1).

*C. elegans* was first isolated from a rich humus soil in Algeria by Emile Maupas at the beginning of the 20th century. About 60 years later Sidney Brenner sampled the N2 strain from a compost heap near Bristol. These anecdotes as well as the sampling of macroscale habitats, such as orchards, riverbanks, and wet shrublands, revealed that *C. elegans* generally prefers habitats with high microbial activity [5,6,7]. Within the environmental habitat, the nematode constitutes the soil biome and the large variety of invertebrate decomposer organisms [8]. *C. elegans* occurs globally, e.g., in parts of Africa, Europe, and the Pacific coast of the US, but could not be located in certain parts of the world such as coastal China [9].

## 3. How Does *C. elegans* Encounter Pollutants?

To highlight the global distribution of pollutants, the focus here is on two examples, the classic neurotoxin mercury and the emerging pollutant manufactured nanoparticles (mNPs). By water column measurements throughout the Atlantic, Pacific, Southern, and Arctic oceans, an ocean inventory of total dissolved mercury was compiled [10]. Soils and sediments likewise represent environmental sinks of mercury deposition and accumulation after human activities, such as artisanal gold mining, metal or cement production, coal-burning, and fossil fuel combustion [11,12]. Environmental flows of emerging pollutants, such as manufactured nanomaterials, have been modelled for Europe, Switzerland, and the US [13,14,15]. Nanomaterial concentrations and their dynamics were predicted for the environmental compartments surface waters, sediments, and soil, allowing for the concept that *C. elegans* represents a real-life target organism of particulate pollutants [16] (Figure 1).

Silica NPs represent nanomaterials that are manufactured, used, and distributed on a large scale [3]. Probabilistic modelling of the flows of nano silica includes wear off from products (e.g., car tires, paints, seed coating) to technical compartments, such as waste treatment plants or directly into the environmental compartments air, soil, surface waters, and sediments [2]. In the European Union (EU) the predicted median concentrations of nano silica in surface waters are in the ng/L range. The predicted median annual increase of these mNPs in sediments and sludge-treated soil is modelled between 420 and 430 μg/kg. Thus, probabilistic scenarios where *C. elegans* may be exposed to nano silica are sediments along river banks, soil along motorways, and agricultural areas, especially those that are fertilized with sewage sludge. As nanomaterials are taken up by plants [17,18], including crops, *C. elegans* may be exposed to mNPs in microbe-rich habitats of plant storage or rotting by trophic transfer, e.g., ingestion of food. Notably, this pathway of mNP-exposure is simulated in the laboratory by the addition of live bacteria to culture media [19,20,21,22,23,24]. 

The modelling of nanomaterial flows is very helpful to envision environmental uptake scenarios for the nematode roundworm *C. elegans*. However, many knowledge gaps remain concerning the concentrations of mNPs that induce biological responses in the environmental habitats of the nematode. Currently, reliable definitions of environmentally relevant lowest observed effect level (LOEL) concentrations are not available. It likewise has to be considered that the microbe rich habitats that are preferred by *C. elegans* may differ considerably between compost heaps, orchards, other agricultural areas, and shrublands. Moreover, non-chemical environmental factors such as temperature most likely play a role in the bioavailability and biological responses of pollutants (Figure 1).

## 4. The Animal Model *C. elegans*

While we acknowledge that analysis of invertebrates such as *C. elegans* in the laboratory can only simulate conditions of environmental exposure scenarios, experimental work with the animal model is extremely powerful for the characterization of biological responses. Culture of *C. elegans* in the laboratory is easy and cost-effective. The small (~1 mm) and transparent worm is amenable to cutting-edge imaging and genome editing-based technologies [25,26]. Gene expression can be investigated by forward genetic screens. Simple feeding of *C. elegans* with bacteria that express double-stranded RNA enables the deletion of gene expression. Concerning development, the entire cell lineage is known from one cell to the 959-celled adult. Despite this simplicity, complex questions can be interrogated. In fact, fundamental principles underlying complex biology are tractable and conserved in this simple model organism.

Research on the function of the worm’s nervous system includes cellular, developmental, and behavioral neuroscience. The neural connectivity map and complete wiring program of *C. elegans* has been characterized. *C. elegans* contributes to our understanding of axon guidance [27] and the role of ultrafast endocytosis in synaptic transmission [28]. Reporter worms enable the examination of single neurons in the context of a living behaving animal. Thus, the worm represents an in vivo cell biology system for the investigation of fundamental questions in neurobiology.

**Figure 2:**
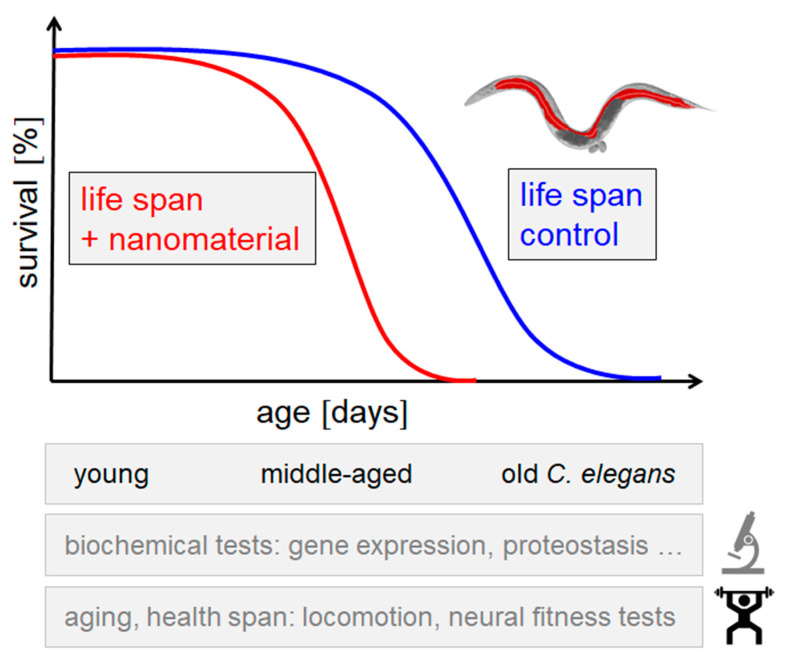
A combination of aging research and toxicology applies life span-resolved investigation of the biological response to nanomaterials. The life span can be determined in unexposed (life span control) vs. adult hermaphrodite *C. elegans* exposed to nanoparticles (+nanomaterial). Biochemical and behavioral analyses can be performed in young, middle-aged and old *C. elegans*.

The worm’s short life span as an adult hermaphrodite of 2–3 weeks is optimal for aging research. Downregulation of insulin signaling was identified as a regulator of longevity in *C. elegans*, and the whole pathway shown to be conserved in the fruit fly *Drosophila melanogaster* and mammals [29]. Due to its tractability in aging and neurobiology research, models of age-related diseases have been developed in the worm for neurodegenerative aggregation disorders, such as Alzheimer’s (AD) and Parkinson’s disease (PD). Moreover, *C. elegans* emerges as an animal model in toxicology. Among other, systems biology approaches have led to the understanding of the organization of a transcriptional circuit that is activated to protect the animal from toxic metabolites [30]. A recently developed combination of aging research and toxicology applies a life span-resolved investigation of the biological response to pollutants (Figure 2).

Assays that measure the life span of *C. elegans* in 96 well microtiter plates [31] were used to investigate toxic effects of manufactured nanomaterials during the entire adult life of the worm [24]. Notably, life span experiments on 96 well microtiter plates and cultivation in liquid S-medium with ad libitum food (*E. coli*) extended the longevity of *C. elegans* in comparison to similar culture conditions on solid agar plates [24]. This suggested that liquid culture minimizes cultivation stress and is therefore optimally suited to characterize the biological response after exposure to toxins. The biological response to mNPs was analyzed by the life span-resolved observation of behavioral or biochemical phenotypes and system biology approaches [23,24]. It was demonstrated that certain nanomaterials, such as nano silica and nano silver, (i) reduced longevity of wild type (N2) *C. elegans*, (ii) induced neurodegeneration of single serotonergic neurons, (iii) promoted amyloid formation in nucleoli of intestinal cells, and (iv) accelerated the neuromuscular defects of locomotion and reproduction. The analysis of protein aggregation by mass spectrometry showed induction of an aggregome with elevated levels of proteins involved in protein homeostasis, such as ribosomal proteins, cellular stress response, and components of proteasomal proteolysis [23,32]. Comparison of pollutant aggregomes revealed that classical neurotoxins such as mercury and the emerging neurotoxin nano silica corrupt the same resilience pathways that are targeted in aging worms [33,34]. Nine super aggregator proteins were identified in mercury, nano silica, and age-induced *C. elegans* aggregome networks that are all linked to human diseases. The methyltransferase fibrillarin (fib-1) is a prominent autoantigen in systemic autoimmune diseases and myopathies, the ortholog of heat shock protein hsp-6 plays a role among other in neurodegenerative diseases such as PD, and the ribosomal protein L7 (rpl-7) is associated with anemias and the autoimmune disease systemic lupus erythematosus [34].

## 5. Contribution of *C. elegans* to the Exposome and One Health

The concept of the exposome combines diverse and dynamic chemical, biological, and physical stresses in the environment and the respective induction of physiological responses [35]. Pollutant effects are viewed as the consequence of interactions between multiple chemical stressors and non-chemical environmental factors, e.g., climate, temperature (Figure 1). All factors that constitute the exposome naturally influence human, animal, and environmental health, which is brought together under the concept of One Health [1,35]. Intact, healthy ecosystems are viewed as the provider and prerequisite of animal, plant, and human health. Thus, a major goal of current toxicology is to characterize the role of the environment in disease by monitoring exposure scenarios, interrogation of the biological response to pollutants, and identification of causal pathways.

A natural contribution of *C. elegans* to the exposome is characterization of the biological response to pollutants in the animal model, since it enables systematic investigation of different organs and disease models. Comparative studies between untreated and pollutant-exposed worms allow to interrogate gene expression, the role of molecular pathways, the function of the gut and uptake of nutrients. The effects of neurotoxins on the nervous system can be analyzed in transparent reporter worms with respect to morphology, neurotransmission, and neuromuscular function [27,36]. Simultaneous microscopical observation is possible from cell biology, e.g., neurodegeneration of single neurons, to the behavioral end point, e.g., locomotion deficits [37]. Due to its short life span, the biological response to pollutants can be investigated throughout the entire adult life of *C. elegans* in young, middle-aged, and old worms [24]. This strategy particularly enables the identification of age specific vulnerabilities and low dose effects, which represent current topics in exposome research.

*C. elegans* is suitable for the characterization of the biological response to pollutants that are subject to oral uptake [38]. The intestinal epithelium of *C. elegans* represents an informative model for intestinal epithelia of higher organisms, including mammals, since the basic cellular structure of the intestine as well as the composition of nutrient receptors is highly conserved between invertebrates and mammals [39]. The nematode covers basic nutritional pathways. Consistent with this, it was shown that nano silica interfered with the OPT-2/PEP-2 dipeptide transporter, disturbed the peptide metabolism, and reduced the growth of adult worms [40,41]. Such information may feed the exposome and motivate interrogation of malnutrition as adverse biological response to other pollutants, e.g., microplastics, and in higher organisms.

In addition to age specific vulnerabilities or low dose effects current topics of exposome research include investigation into mixtures of toxins as well as the contribution of non-chemical factors. Here, another feature of the animal model *C. elegans* is very helpful, namely its ease of cultivation in microtiter well plates. In a seminal high-throughput study, Jung et al. analyzed 20 different manufactured nanoparticles in *C. elegans* concerning multiple endpoints, such as food consumption, body length, locomotion speed, and life span [21]. Various degrees of toxicity were attributed to (i) different nanoparticles and their biophysical properties, such as size, shape, and surface chemistry, or (ii) the conditions of the exposure. A non-chemical stressor that recently has attained more attention is ambient temperature. Consistent with this, it was shown that until middle age, worms cultivated at 15 °C performed better in swimming tests in comparison with their counterparts that lived at 20 °C or 25 °C [42]. Such interrogation of the *C. elegans* health span provides a promising approach to characterize the biological response to pollutants in combination with the non-chemical exposure factor temperature and identify vulnerable, temperature sensitive age-groups.

Generally, the nervous system of *C. elegans* is equipped with a set of thermosensory neurons (AFD, AWC, ASI, and ASJ) that sense the temperature of the environment. AFD plays a major role to inform between ambient temperature and *C. elegans* behavior. In a cultivation window of 15–25 °C increasing temperatures affect the worm’s metabolism, e.g., proteostasis, trigger a heat shock response, and promote translation by the initiation factor (eIF)-4E [43,44,45]. Since wild type (N2) *C. elegans* have a longer mean life span at 15 °C and a shorter mean life span at 25 °C [46], it will be important to further elucidate the biological response to chemical stressors such as nanomaterials and the non-chemical stressor temperature: that is, the interactions between pollutants and organismal metabolism, the nervous system, health, and longevity at the level of gene expression as well as molecular pathways.

## 6. From Worms to Men

**Figure 3:**
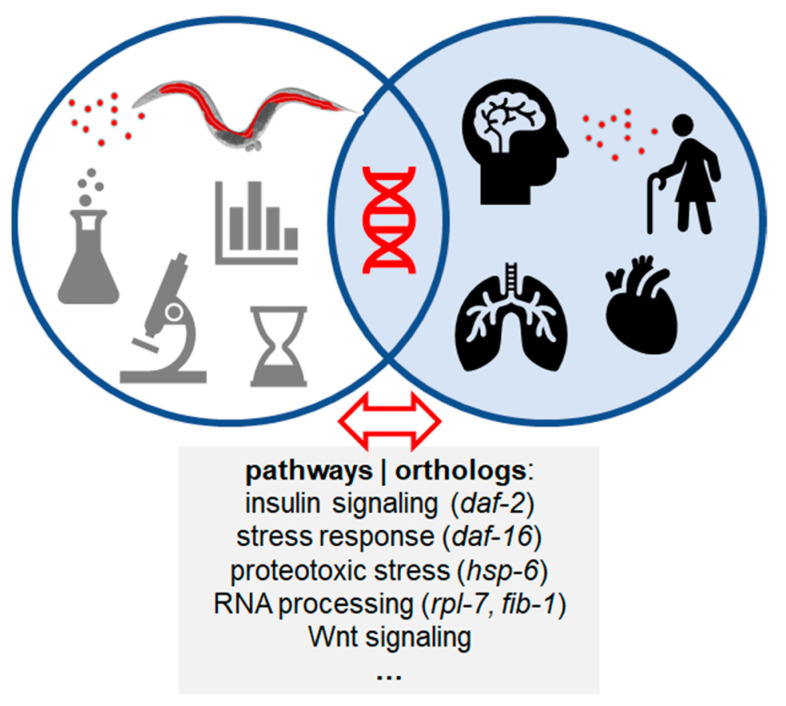
Characterization of gene expression (intersection, DNA double helix, red) by transcriptomics or proteomics in the model organism *C. elegans* (left set) and whole genome association studies (WGAS) in human cohorts (right set) exposed to nanomaterials (red dots). *C. elegans* orthologs of human genes play a role in aging and neurodegenerative, cardiovascular or pulmonary diseases (right set). *Daf-2*, insulin-like growth factor 1; *daf-16*, FOXO transcription factor; *hsp-6*, heat shock protein 6; *rpl-7*, ribosomal protein L7; *fib-1*, fibrillarin; Wnt, Wnt signaling pathway.

The natural question that arises concerns the transferability of the biological response in the invertebrate nematode *C. elegans* to higher order species including humans. Notably, adult *C. elegans* possess organs such as intestinal epithelia and a nervous system with a cell biology and function that is simple but provides sufficient resemblance to human counterparts for a thorough investigation of basic biology. Decades of research using the model organism *C. elegans* has been successful to elucidate basic biological processes, such as apoptosis, cell polarity, regulation of gene expression, metabolism, and aging. It turned out that there is a remarkably high level of conservation of cellular and molecular pathways between the invertebrate nematode and mammals (Figure 3).

Briefly, 60–80% of human genes have an ortholog in the *C. elegans* genome, including 40% of genes that are known to be associated with human diseases [47,48]. A comparison between model organisms and humans revealed approximate numbers of protein coding genes for *Homo sapiens* (19,000), *Mus musculus* (22,000), *Drosophila melanogaster* (14,000), and *Caenorhabditis elegans* (22,000). Thus, the worm represents a powerful model organism to validate the causal-effect role of environmental risk factors identified in human correlative studies on complex behaviors associated with aging or development, and to investigate the exact molecular mechanisms behind it (Figure 3; [49]). Among the signal transduction pathways prevalent in *C. elegans* are the RTK/Ras, the Wnt, the Smad, the Notch pathway, and others. Additionally, neural signaling (calcium, neuropeptides, neurotransmitters) and insulin signaling pathways have been uncovered in the worm, which represents an attractively simple model to interrogate complex and evolutionary conserved pathways [27,29,34]. The *C. elegans* nervous system is used increasingly as a model for the cellular basis of human diseases.

Many models for neurodegenerative diseases have been developed in *C. elegans*. The Alzheimer’s disease (AD) models include reporter worms for aberrant protein aggregation of amyloid-β peptide (Aβ) and tau proteins, whereas the Parkinson’s disease (PD) reporters show amyloid-like aggregation of the disease marker protein alpha-synuclein [50,51,52]. Besides interrogation of the molecular pathways of amyloid protein aggregation and neurodegeneration, the *C. elegans* disease models were developed as tools for the screening of neuroprotective compounds. Economical medium- to high-throughput screens have been established that identify drugs and gene targets for therapy and treatment of PD [53].

## 7. Neurodegeneration and Neuronal Death in *C. elegans* PD Models

A hallmark of PD is the neurodegeneration of dopaminergic neurons in specific regions of the brain, such as the substantia nigra’s pars compacta, and resulting dopamine deficiency in basal ganglia [54]. The cause of this specificity and molecular pathways of neuronal death are largely unknown. Here, the simple dopaminergic system of *C. elegans* that consists of eight dopaminergic neurons allows for detailed investigations on how neurons actually die during PD, including a possible role of pollutants. In transgenic nematodes that express the PD-associated and fibrillation-prone protein alpha-synuclein [55], mitochondrial damage induced the translocation of transcription factor ATFS-1 from the cytoplasm to the cell nucleus as well as a prolonged activation of the mitochondrial unfolded protein response (UPR)^MT^ [56]. Both alpha synuclein and ATFS-associated deregulation of UPR^MT^ homeostasis acted together to accelerate the non-apoptotic death of dopaminergic neurons that occurred without a significant role of the apoptosis gene *ced-4* [57]. Thus, overactivation of the UPR^MT^ resulted in the dysregulation of proteostasis in vulnerable cells, e.g., dopaminergic neurons.

In response to trauma, axons usually follow a degenerative process composed of beading, thinning, fragmentation, and clearing of axonal debris, e.g., axonal retraction to the neuron’s soma [58]. Nichols et al. showed that PLM axons injured by laser ablation degenerate, regulated by the apoptotic engulfment machinery involving conserved genes *ced-1*, *ced-6*, *ced-7,* and *nrf-5* [59]. However, it remains to be demonstrated if this pathway plays a role in *C. elegans* PD models and the degeneration of dopaminergic neurons. Notably, recent work indicates that mitochondrial dysfunction can trigger programmed axon death independent of physical trauma and play a role in neurodegenerative diseases that involve early axon loss, including PD and peripheral neuropathies [60].

Certain pollutants including manufactured nanomaterials likewise induce molecular cascades of neurodegeneration. It has been shown in dopaminergic and serotonergic neurons that manganese, methylmercury, or nano silica induce protein aggregation in axons and disturb trafficking of cargoes, such as mitochondria and neurotransmitters, to the synapse [23,61,62,63]. Due to a well-characterized neural wiring, the loss of neuronal functioning can be monitored in *C. elegans* by quantification of neuromuscular behaviors under the control of respective dopaminergic, GABAergic or serotonergic neurons [64]. For future research, it will be important to learn more about the role of molecular pathways and the timing of the neurodegeneration cascade. Which molecular steps in the cascade are specifically vulnerable to pollutant effects and what is the temporal schedule?

## 8. Investigation of Pollutant Effects in Wild *C. elegans*

The environmental one health paradigm can also be tested by an innovative approach that brings the field into the lab [65]. The isolation and cultivation of nematodes from wild habitats represents a promising strategy to bridge environment and laboratory. This approach aims at molecular characterization of *C. elegans* or other free-living soil nematodes that are sampled from environmental habitats and directly taken into culture [66]. Subjecting the sampled nematodes to single worm proteomics [67] enables characterization of the gene expression in wild nematodes and the usage of molecular pathways. The function of the wild nematodes’ nervous system can be assayed by neuromuscular phenotyping of locomotion, such as crawling, swimming or swim-crawl transitions [53]. Determination of the biological response locomotion, e.g., ‘no observed effect levels’ (NOELs) and ‘lowest observed effect levels’ (LOELs) in nematodes isolated from the environment has the potential to close our gap of knowledge concerning environmentally relevant concentrations of pollutants and respective risk assessment. While many of the conventional laboratory methods require a certain degree of adaptation, gaps between environmental exposure and biological responses may be addressed by comparative investigations between *C. elegans* or other soil nematodes sampled from unpolluted vs. polluted habitats [65].

## 9. Future Goals and Perspectives

While the cultivation of *C. elegans* in micro titer plates and liquid media allows for medium to high throughput investigation of biological responses to pollutants, one has to acknowledge that more than 350,000 chemicals are in production and usage [1]. The pace of introducing new nanomaterials and new nanotechnological applications clearly exceeds the potential of research into their safety. The natural question is whether and how toxicological research can keep up with identification of possible adverse effects on animals, plants, and humans, e.g., the environment. Moreover, real-world exposures include aging of chemicals in the environment, respective degradation products of chemicals, their complex mixtures, and nonchemical stressors such as temperature [35].

A combination of the exposome and one health concepts may turn out extremely helpful to meet this challenge. With the development of computational strategies for the compilation of exposome data, biological pathways may be discovered at an as yet unprecedented pace. Common pathways of vulnerabilities against pollutants, but likewise resilience, await characterization. This requires an interdisciplinary approach that brings together, among others, research in invertebrates such as *C. elegans* and epidemiology, e.g., research in pollutant-exposed human cohorts [49]. Other developments seem promising to further expedite the elaboration of exposome and one health concepts. Cultivation of model organisms such as *C. elegans* on microtiter plates provides the inherent potential for the automation and generation of big data. An increasing number of open data platforms are being developed. For example, open-source automated assays and data bases were introduced for amyloid like proteins and *C. elegans* models of neurodegenerative aggregation diseases, such as AD and PD [68,69]. Similar concepts of toxicological research in simple model organisms, automation, and open databases may provide the key to safe nanotechnology.

## Data Availability

Not applicable.
